# A simple and versatile interface to feed analogue data from the output of analytical instruments to a BBC microcomputer

**DOI:** 10.1155/S1463924687000294

**Published:** 1987

**Authors:** L. Ebdon, J. I. Garcia Alonso, S. J. Hill, A. Hopkins

**Affiliations:** 1 Department of Environmental Sciences Plymouth Polytechnic Drake Circus Devon Plymouth PL4 8AA UK; 2 Department of Analytical Chemistry University of Oviedo Spain


**Journal of Automatic Chemistry**/*Journal of Clinical Laboratory Automation*, **Vol. 9, No. 3 (July–September 1987), pp. 132–134**

### A simple and versatile interface to feed analogue data from the output of analytical instruments to a BBC microcomputer

**L. Ebdon†, J. I. Garcia Alonso*, S. J. Hill and A. Hopkins**

*Department of Environmental Sciences, Plymouth Polytechnic, Drake Circus, Plymouth, Devon PL4 8AA, UK*

*An interface is described to convert analogue data from the recorder or other output of a typical analytical spectrometer, or other instrument, to an amplified form suitable for feeding to the ADC of a microcomputer (BBC B+). The interface is simple and easy to construct, avoids complicated software, and can be used for a variety of applications. Excellent linearity was observed in transformed data and the interface is particularly suited to handling transient signals. Examples of its use in atomic absorption spectrometry, solution spectrophotometry and high performance liquid chromatography are given.*

### Introduction

In spite of the extraordinary development of computers and computer-assisted analytical instrumentation there is still a widespread use of chart-recorders to record analytical data. In a brief survey of leading journals in analytical chemistry published during 1986 we observed that chart-recorders were used in over 40% of the papers with high performance liquid chromatography (HPLC) applications and in over 80% of the papers using flow-injection analysis (FIA). This is surprising given the limited data-handling facilities of chart-recorders, the time-consuming and manual nature of the interpretation of results, the inherent noise problems and difficulties in signal filtering and the ready availability of inexpensive personal computers, competitively priced compared to chart-recorders. While there has been considerable growth in the use of purpose-designed computing integrators for chromatography, the lack of versatility inherent to many commercial integrators and computer-controlled instruments makes the development of new analytical techniques which use discrete sample introduction difficult. For example, the electrothermal vaporization of analyte into the inductively coupled plasma (ICP) or microwave induced plasma (MIP) generates a discrete analyte emission signal not always totally suitable for a chart-recorder because of the required speed of response, yet chart-recorders are still used in about 60% of applications.

In recent investigations into the development of a new interface for directly coupled high performance liquid chromatography – flame atomic absorption spectroscopy (HPLC–FAAS), based on a series of rotating Pt spirals, which transport discrete aliquots of sample to a flame atomizer [1–3], it was found that the use of a chart-recorder offered only limited data handling facilities. Ideally transient non-atomic absorption peaks arising from introduction of the spirals into the flame would be eliminated, deviations in the background as the flame gases are disrupted corrected, small variations in response obtained from individual spirals compensated for and peak heights and areas computed . To achieve this aim the analogue recorder output from an SP-9 Atomic Absorption Spectrometer (Pye Unicam Ltd, Cambridge, UK) was interfaced to a BBC B+ microcomputer (Acorn Computers Ltd, Cambridge, UK). In addition, control of various instrumental parameters, such as the rotation of the stepper motor [2], was incorporated into the software. We believe that this simple, cheap and reliable interface has many useful applications in those fields where the use of basic chart-recorders limits the value of the data obtained.

### Interface characteristics

The analogue voltage from the recorder output of the SP-9 spectrometer (10 mV) was amplified 100 times and the signal was then fed to the analogue-to-digital converter (ADC) of the BBC microcomputer which can convert signals up to 1·8 V into digital values between 0–4095. Only one channel was used in the ADC (up to four channels are available) facilitating 100 conversions/s. The digital values can be read and stored by a simple BASIC program using the X = ADVAL(1)/16 command.

Figure 1 shows the electronic circuit of the interface. Power supply (+5 V) was obtained from the computer via the analogue input port. The circuit consists of a high impedance MOS/FET operational amplifier, connected in a non-inverting mode. Gain = $(R_2 + R_1)/R_2$.

Maximum output ($P_1$) is limited to half the supply voltage less 0·1 V, a potential divider network is used to reduce this to a maximum of 1·7 V ($P_2$) in order to protect the input to the ADC.

The performance of the interface was evaluated with the atomic absorption spectrometer. Figure 2 shows the excellent linearity observed between absorbance and

†*Author to whom correspondence should be addressed.*
**On leave from Department of Analytical Chemistry, University of Oviedo, Spain.*

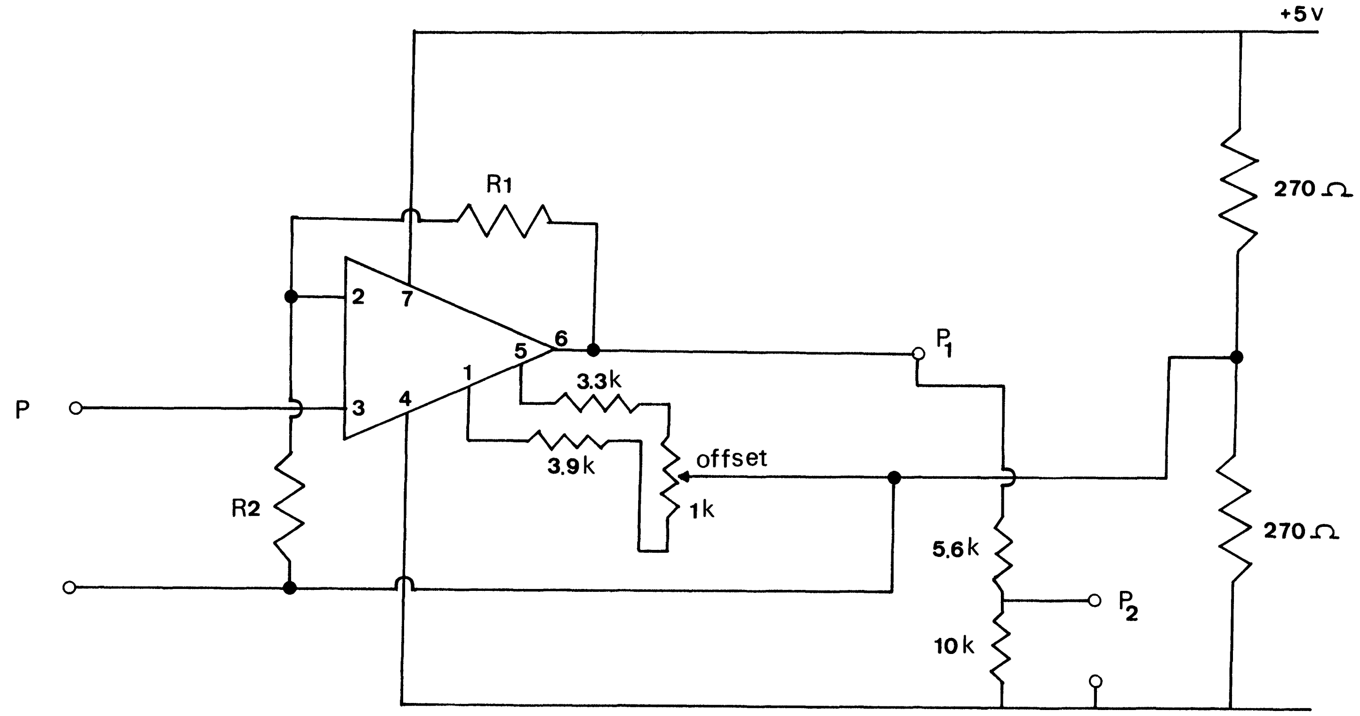

*Figure 1. Circuit diagram for the interface.*

transformed digital values both with and without scale expansion. The electronic noise of the interface was determined to be constant at ±2 units between levels of 100 and 700 units. Figure 3 shows the transient atomic absorption peaks obtained for mercury, cadmium, and lead with the HPLC-FAAS coupling. As can be observed, the interface has no difficulty in following the peak profiles, even for rapidly atomized elements such as mercury (i.e. very transient peaks).

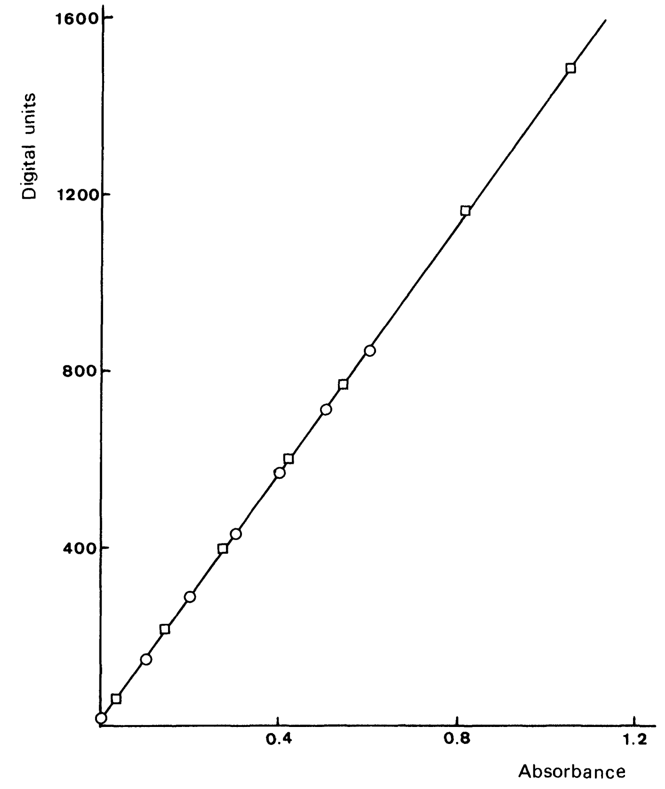

*Figure 2. Linearity of the response of the interface shown in the conversion of digital units to absorbance (○) direct absorbance; (□) × 8.3 scale expansion.*

### Applications

Apart from possible educational applications, the new interface can be used in many different fields, including collection of spectrophotometric and fluorimetric spectra

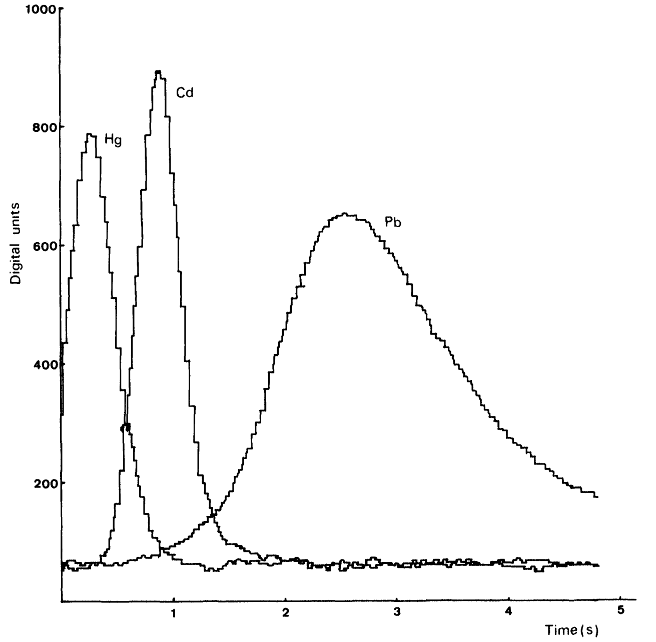

*Figure 3. Transient atomic absorption peaks for Hg, Cd and Pb from a platinum spiral introduced into the flame as displayed via the interface and computer on a visual display unit.*

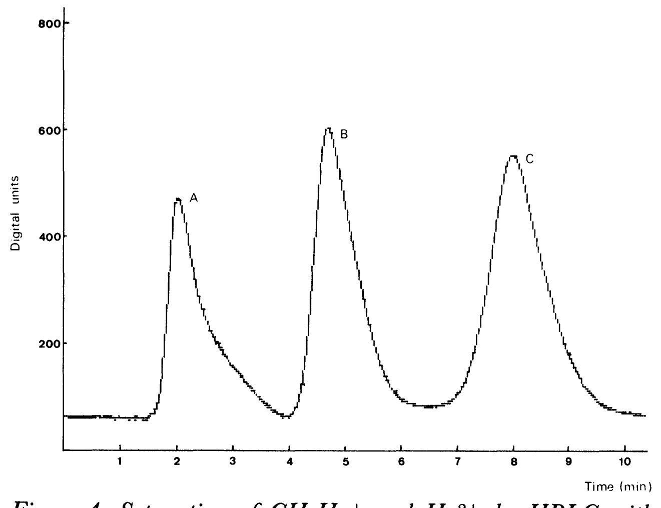

*Figure 4. Separation of $CH_3Hg^+$ and $Hg^{2+}$ by HPLC with spectrophotometric detection at 475 nm. Eluent 85% methanol with $2 \times 10^{-4}$ M EDTA and $6 \times 10^{-3}$ M acetate buffer at pH = 4·1. Flow 0·4 ml $min^{-1}$, column 250 × 2 mm Shandon ODS-hypersil 5 um particle size. Injection (20 µl) as dithizone complexes in methanol. (A) Dithizonw, (B) Methyl mercury dithizonate, (C) Mercury (II) dithizonate.*

when numerical treatment of the data is necessary, and the collection, storage and manipulation of chromatographic data. Figure 4 shows a chromatogram obtained from the separation of $CH_3Hg^+$ and $Hg^{2+}$ as dithizone complexes [4] on a minibore (2 mm id) column packed with ODS, 5 µm particle size. The output from the spectrophotometric detector (set at 475 nm) was fed to the BBC micro computer and the signal integrated over 1 s. Once the chromatogram has been obtained and stored on disk, new possibilities are open for the manipulation, quantification and comparison of chromatograms. Other possible applications are numerical treatments of 'FIAgrams' and in coupled techniques where the data are obtained as transient peaks (for example FIA-preconcentration-AA/ICP, electrothermal vaporization into plasmas, coupled chromatography-spectrometry).

Further information on the interface and computer programs for the BBC can be obtained from the authors.

### Acknowledgement

Dr J. I. Garcia Alonso wishes to thank the FICYT (Oviedo, Spain) for the provision of a post-doctoral grant.

### References

1. Hill, S., Ebdon, L. and Jones, P., *Analytical Proceedings*, **23** (1986), 6.
2. Ebdon, L., Hill, S. and Jones, P., *Journal of Analytical Atomic Spectrometry*, **2** (1987), 205.
3. Ebdon, L., Hill, S. and Jones, P., *Analyst*, **112** (1987), 437.
4. Langseth, W., *Analytica Chimica Acta*, **185** (1986), 249.

### 39TH PITTSBURGH CONFERENCE AND EXPOSITION ON ANALYTICAL CHEMISTRY AND APPLIED SPECTROSCOPY

*22 to 26 February 1988: New Orleans, Louisiana, USA*

The technical programme for the 1988 Pittcon will consist of 30 symposia and *c.* 900 contributed papers. Poster sessions will supplement oral presentations on the latest developments in analytical chemistry and applied spectroscopy.
The progamme committee will consider 500-word abstracts for inclusion from now to 3 August 1987. Papers are welcome in the following areas:

Atomic absorption spectroscopy
Automated analysis
Biochemical analysis
Biomedical pharmaceutical
Classical chemical analysis
Clinical chemistry
Computer applications
Countercurrent chromatography
Electrochemistry
Emission spectroscopy
Field flow fractionation
Flow injection analysis
Fluorescence-luminescence
Gas chromatography
Gel permeation chromatography
Industrial hygiene
Infrared spectroscopy
Ionchromatography
Laboratory robotics
Laser applications in spectroscopy
Liquid chromatography
Mass spectrometry
Near infrared
New instrumentation
Nuclear magnetic resonance spectroscopy
Particle size analysis
Pesticide analysis
Plasma emission spectroscopy
Polymer analysis
Process stream analysis
Raman spectroscopy
Selective ion electrodes
Supercritical fluid chromatography
Surface analysis
Thermal analysis
Thin layer chromatography
Toxicological analysis
Trace analysis
UV-VIS spectrophotometry
X-Ray diffraction/fluorescence spectroscopy

*Abstracts should be sent to Mrs Alma Johnson, Program Secretary, 12 Federal Drive, Suite 322, Pittsburgh, Pennsylvania 15235, USA.*

